# Capivasertib-induced polymorphous lichenoid exanthem: Report of a novel AKT kinase inhibitor-related drug eruption

**DOI:** 10.1016/j.jdcr.2025.05.022

**Published:** 2025-06-14

**Authors:** Raj P. Fadadu, Albert Lee, Brian Hinds, Charisse Orme

**Affiliations:** Department of Dermatology, University of California San Diego, San Diego, California

**Keywords:** breast cancer, drug, drug eruption, oncodermatology, oncology, rash

## Introduction

Cancer therapies that inhibit phosphoinositide 3-kinase-protein kinase B- (PI3K-AKT)-mammalian target of rapamycin signaling are expanding in usage and development, as this pathway is frequently overactivated in malignancies of various organs. The first-in-class oral AKT kinase (protein kinase B) inhibitor, capivasertib, received Food and Drug Administration approval in November 2023 for treatment of patients with advanced hormone receptor-positive, human epidermal growth factor receptor 2-negative breast cancer based on the phase III CAPItello-291 clinical trial.^1^^,^^2^ Cancer therapy often causes cutaneous adverse reactions, but there is a lack of scientific literature describing clinical and histopathological characteristics of skin eruptions associated with AKT inhibitors despite the high prevalence of cutaneous reactions experienced by patients on capivasertib therapy in clinical trials. We present a case report of a diffuse, pruritic, polymorphous lichenoid rash associated with capivasertib.

## Report of a case

A 68-year-old woman with progressive metastatic ductal carcinoma refractory to multiple cancer therapies initiated oral capivasertib monotherapy on a regimen of 400 mg twice daily for 4 days, followed by 3 days off. Two weeks later, she developed an itchy, nonpainful rash on the right leg that spread to a generalized distribution. She noted several hours of sun exposure on the prior day. Additional symptoms included oral dysesthesia, an oral ulcer, and eye grittiness, but she denied any new symptoms of dysphagia, diarrhea, joint pains, fatigue, or vision changes.

**Fig 1:**
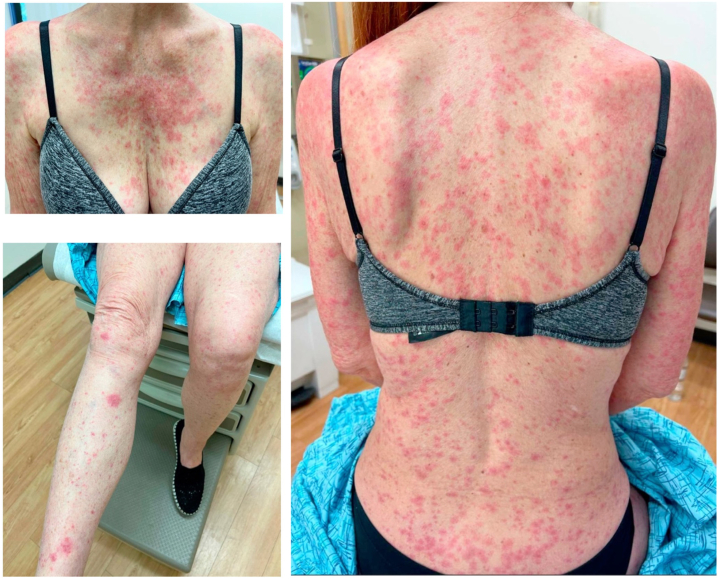
Clinical photos of cutaneous adverse reaction to capivasertib.

**Fig 2:**
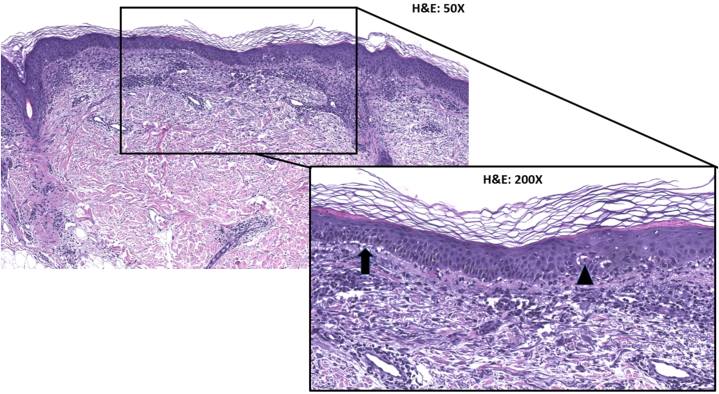
Histopathologic examination of rash biopsy demonstrated acute atrophic lichenoid interface dermatitis with foci of vacuolar interface reaction (*arrow*), singly necrotic keratinocytes (*triangle*), and no evidence of mucin in the dermis. Stain: hematoxylin and eosin. Original magnification: 50×. Inset magnification: 200×.

On physical examination, discrete erythematous papules, macules, and plaques with mild scaling were scattered across the neck, trunk, and extremities (Fig 1). She had confluent erythematous facial patches and a well-defined erythematous ulceration on the mucosal lip. Nikolsky sign was negative. Vital signs were stable compared to prior, and recent serum laboratory studies revealed no acute findings. Two punch biopsies were performed on the left arm and central back. Histopathologic examination featured an atrophic interface dermatitis with patchy band-like lymphocytes, foci of vacuolar interface reaction, and singly necrotic keratinocytes (Fig 2). The clinicopathologic findings were consistent with drug reaction to capivasertib, classified as a grade 3 cutaneous adverse reaction. In consultation with oncology, she was counseled to stop capivasertib, start an oral prednisone taper (beginning with 30 mg daily, around 0.5 mg/kg/day), and start applying topical triamcinolone ointment. The eruption resolved in 10 days with mild postinflammatory pigmentary alteration, and her oral ulcer and sensation of eye grittiness resolved within 3 weeks. Due to this eruption, she was switched to another cancer treatment plan.

## Discussion

Capivasertib is an adenosine triphosphate-competitive AKT kinase inhibitor that was commonly associated with cutaneous adverse reactions in clinical trials, although the specifics of this skin toxicity have been largely undefined. In the phase II trial of capivasertib monotherapy with 41 participants, 100% of participants who received 480 mg twice daily dosing for 4 days followed by 3 days off experienced a maculopapular rash.^3^ Safety data from the phase III trial showed that 38% (*N* = 135) of participants in the treatment group developed a rash, of which 33% (*N* = 43) were classified as grade 3.^1^ Cutaneous reactions (58%, all-grade rashes) were more common in a subanalysis of patients who had AKT pathway-altered tumors.^4^ Nineteen percent of patients with skin reactions required systemic steroids. Of note, rashes encompassed a variety of reported morphologies, including papular, eczematous, erythema multiforme-like, and urticarial. The underlying pathogenesis may relate to skin hypersensitivity reaction to the medication or aberrant keratinocyte proliferation and differentiation due to inhibition of PI3K-AKT signaling, as seen in skin reactions to PI3K inhibitors.^5^, ^6^, ^7^

Dermatologists should recognize that a skin reaction to capivasertib can present with variable clinical and morphological features. They should also evaluate for mucosal involvement, such as mucositis or oral ulcers in the case of this patient, although these symptoms were less common compared to cutaneous symptoms in CAPItello-291.^1^ One other case report of cutaneous toxicity to capivasertib has been previously published.^8^ In this report, one patient received capivasertib monotherapy and the other patient received dual therapy with fulvestrant. For both patients, the rash morphology was erythema multiforme-like, which supports the multiple possible clinical presentations of capivasertib-associated skin reactions. Both eruptions occurred within a similar time frame after drug initiation compared to this case—1 to 2 weeks. This is concordant with results from a study of patient-reported outcomes from CAPItello-291, in which presence of rash and severity of itchy skin were highest in the treatment group at day 15 after cycle 1 of treatment.^9^ Histopathology for our patient demonstrated evidence of dyskeratosis and an interface dermatitis that was more lichenoid, in contrast to the prior case report.^8^ More data are needed to elucidate if there are specific histologic characteristics for capivasertib-associated exanthems. The density of the lymphocytes in our case exceeded that of traditional viral or drug-induced morbilliform exanthem. Increased sun exposure may have been a risk factor that contributed to rash development in this patient, which has not been previously reported.^8^ Lesions were present on both sun-exposed and sun-unexposed skin. Certain immune checkpoint therapies like programmed cell death protein 1 inhibitors can cause cutaneous photosensitivity reactions,^10^ but there is less evidence of this for medications that inhibit the PI3K-AKT pathway. Further study is needed to determine the role of sun protection in reducing risk of capivasertib-associated skin toxicity.

It is important for dermatologists to communicate with patients’ oncologists to manage cutaneous symptoms of immune checkpoint inhibitors within the context of the overall plan for cancer therapy. There are many risk factors and management options to consider that are unique to each patient. In the case of mild eruptions, it is reasonable to begin use of topical medications and continue cancer therapy at the same dose. Alternatively, following the resolution of a moderate-severe eruption, a retrial of the cancer medication at a lower dose could be considered. In our patient case, due to the severity of symptoms and other options for her cancer therapy, a joint decision was made with the patient to stop capivasertib, not pursue retrial of the medication, and start a new cancer therapy.

Capivasertib and other AKT inhibitors are being studied in clinical trials for multiple solid tumors, including prostate cancer and meningiomas, and may become more common in oncologic clinical practice. The rapidly expanding selection of targeted drug therapies has ushered in an era of precision medicine where oncologists can leverage patient characteristics, such as tumor mutation status, to select the optimal therapeutic regimen. Dermatologists should recognize the relatively high incidence of polymorphous skin adverse reactions to AKT inhibitors and be ready to help manage them.

## Conflicts of interest

None disclosed.

## References

[1] Turner N.C., Oliveira M., Howell S.J. (2023). Capivasertib in hormone receptor–positive advanced breast cancer. N Engl J Med.

[2] Burstein H.J., DeMichele A., Fallowfield L., Somerfield M.R., Henry N.L. (2024). For the biomarker testing and endocrine and targeted therapy in metastatic breast cancer expert panels. Endocrine and targeted therapy for hormone receptor–positive, human epidermal growth factor receptor 2–negative metastatic breast cancer—capivasertib-fulvestrant: ASCO rapid recommendation update. J Clin Oncol.

[3] Tamura K., Hashimoto J., Tanabe Y. (2016). Safety and tolerability of AZD5363 in Japanese patients with advanced solid tumors. Cancer Chemother Pharmacol.

[4] Dilawari A., Buturla J., Osgood C. (2024). US food and drug administration approval summary: capivasertib with fulvestrant for hormone receptor–positive, human epidermal growth factor receptor 2–negative locally advanced or metastatic breast cancer with PIK3CA/AKT1/PTEN alterations. J Clin Oncol.

[5] Teng Y., Fan Y., Ma J. (2021). The PI3K/Akt pathway: emerging roles in skin homeostasis and a group of non-malignant skin disorders. Cells.

[6] Wang Y., Ma Z., An Z., Zhang Y., Feng X., Yu X. (2023). Risk of cutaneous adverse events in cancer patients treated with phosphatidylinositol-3-kinase inhibitors: a systematic review and meta-analysis of randomized controlled trials. Cancer Med.

[7] Wang D.G., Barrios D.M., Blinder V.S. (2020). Dermatologic adverse events related to the PI3Kα inhibitor alpelisib (BYL719) in patients with breast cancer. Breast Cancer Res Treat.

[8] Keum H., Zhu J.L., Heberton M., Vandergriff T., Dominguez A.R. (2024). Erythema multiforme-like targetoid eruption in two patients treated with capivasertib for metastatic breast cancer. JAAD Case Rep.

[9] Oliveira M., Rugo H.S., Howell S.J. (2024). Capivasertib and fulvestrant for patients with hormone receptor-positive, HER2-negative advanced breast cancer (CAPItello-291): patient-reported outcomes from a phase 3, randomised, double-blind, placebo-controlled trial. Lancet Oncol.

[10] Ellis S., Vierra A.T., Millsop J.W., Lacouture M.E., Kiuru M. (2020). Dermatologic toxicities to immune checkpoint inhibitor therapy: a review of histopathologic features. J Am Acad Dermatol.

